# Temporal eating patterns: a latent class analysis approach

**DOI:** 10.1186/s12966-016-0459-6

**Published:** 2017-01-07

**Authors:** Rebecca M. Leech, Anthony Worsley, Anna Timperio, Sarah A. McNaughton

**Affiliations:** The Institute for Physical Activity and Nutrition (IPAN), School of Exercise and Nutrition Sciences, Deakin University, Geelong, Australia

**Keywords:** Chrono-nutrition, Eating occasion, Eating patterns, Latent class analysis, Meals, Meal timing, Snacks

## Abstract

**Background:**

There is some evidence that large energy intakes towards the end of the day are associated with adverse health outcomes, however, studies of temporal eating patterns across the day are rare. This study examines the temporal eating patterns of Australian adults using latent class analysis (LCA), as a novel approach.

**Methods:**

Dietary data (*n* = 2402 men and *n* = 2840 women, ≥19 years) from two 24-h recalls collected during the 2011–12 Australian National Nutrition and Physical Activity Survey were analyzed. LCA was performed to identify distinct temporal eating patterns based on whether or not an eating occasion (EO) occurred within each hour of the day. *F* and adjusted-chi^2^ tests assessed differences in sociodemographic and eating patterns (e.g., meal, snack and EO frequency) between latent classes.

**Results:**

Three patterns, labelled “Conventional” (men: 43%, women: 41%), “Later lunch” (men: 34%, women: 34%) and “Grazing” (men: 23%, women: 25%) were identified. Men and women with a “Grazing” pattern were significantly younger (*P* < 0.001) and a higher proportion were from major cities (*P* < 0.01) and were not married (men only, *P* = 0.01), compared to the “Conventional” and “Later lunch” patterns. The “Grazing” pattern was also characterized by a higher EO frequency (*P* < 0.01) and snack frequency (*P* < 0.001) and consumption of a higher proportion of total energy intake from snacks but a lower proportion of total energy intake from meals (*P* < 0.001).

**Conclusions:**

This study identified three distinct temporal eating patterns in adults that varied by age, EO frequency, snack frequency and energy intake pattern. LCA is a useful approach to capture differences in EO timing across the day. Future research should examine associations between temporal eating patterns and health.

## Background

There is increasing interest in understanding the timing or patterning of food intake or eating occasions (EOs), including meals and snacks, and how this influences health [1–3]. Existing research examining patterning of EOs is diverse, and covers a range of concepts including EO frequency and spacing, meal regularity, timing and skipping or temporal eating patterns [2]. “Temporal eating patterns” refers to the timing, frequency and regularity of food intake or EOs across the day [4]. This emerging field of research on the timing of food intake is known as “chrono-nutrition [4, 5].

Timing of food intake may play an important role in health outcomes given the interplay between timing of food intake, circadian rhythms, physiology and metabolism [5]. Prospective observational studies have found that shift workers have a higher risk of metabolic syndrome [6] and type 2 diabetes [7]. A recent review of ten observational studies also found some evidence of a positive association between evening energy intake and overweight [4]. However, inconsistent approaches used to assess temporal eating patterns and the lack of adjustment for energy intakes at other EOs were noted as limitations of the reviewed studies [4]. Moreover, EO timing has mostly been assessed using arbitrary time-points [8–12]; studies of EO timing across the day are rare [13, 14]. Novel analytic methods that account for all EOs consumed across the day are needed.

Data-driven analytic approaches such as cluster analysis and latent class analysis (LCA) are exploratory tools that can be used to identify distinct, unknown patterns in subpopulations based on a set of observed indicators from multiple layers of data [15, 16]. While these approaches have been previously used to identify dietary [17, 18] and lifestyle behavior patterns [19, 20], few studies have considered timing of EOs to identify behavioral patterns [13, 14]. These approaches also do not require a predefined definition of EO timing and may thus be useful to identify unique temporal eating patterns using data on EOs consumed across the day.

Although few published studies have examined temporal eating patterns, there is evidence to suggest that these patterns vary by country and geographical region [4]. This finding may be explained by sociodemographic and/or sociocultural differences that influence eating habits such as socioeconomic factors, family structure, cultural emphasis on certain meals or working patterns [4, 21–24]. Therefore, using LCA as a novel approach, the aims of this study were to examine the temporal eating patterns of Australian men and women and to evaluate these patterns according to their sociodemographic and eating pattern profile.

## Methods

### Sample and study design

The 2011–2012 Australian National Nutrition and Physical Activity Survey (NNPAS 2011–12) is a cross-sectional, nation-wide survey administered by the Australian Bureau of Statistics (ABS). The survey design and data collection methods have been published in detail previously [25]. Briefly, the survey employed a multistage, probability sampling design of private dwellings, and included 12,153 persons aged 2 years or over (77% response rate). Of these respondents, 9338 were adults aged ≥ 19 years. Person-specific weights, adjusted for probability of selection and non-response, were used to provide estimates relating to the whole population. The *Census and Statistics Act 1905* provides ethics approval for the ABS to conduct the household interview components of health surveys [25].

### Dietary assessment

Dietary data were collected during two 24-h recalls (validated USDA automated multiple 5-pass method), conducted approximately nine days apart [26, 27]. Of the 9338 adult respondents, 6053 (65%) completed both dietary recalls. Information on respondents’ EOs were collected during each 24-h recall. Respondents identified the type of EO and the time when each EO commenced. The EO response options were: breakfast; brunch; lunch; dinner; supper; snack; morning tea; afternoon tea; drink/beverage; extended consumption; other; and don’t know/not determined. The Australian Supplement and Nutrient Database 2011–13 was used to calculate energy and nutrient intakes from all foods and beverages [28]. Dietary information was averaged across the two days of recall to obtain mean estimates of energy intakes and eating patterns.

### Eating occasion timing and spacing

An EO was defined as any occasion where a food or beverage was consumed that contains ≥ 210 kJ and is separated in time from the preceding and succeeding EO by 15 min. This EO definition has been previously shown to be a better predictor of overall energy intakes and adiposity, than definitions with no energy criterion or larger time intervals [29, 30]. EOs across the day were examined and a binary variable was created to indicate whether or not an EO had occurred within each hour of the day. The clock time of when each EO commenced was also used to calculate the mean time between EOs. Therefore, estimates relating to spacing between EOs were independent of EO duration.

### Eating patterns

Meals and snacks were classified based on participant self-report, consistent with previous research [2]. There is currently no consensus on which approach is best for classifying meals and snacks [29]. However, a recent study found little difference in predicting variance in total energy intake when meals and snacks were based on either self-report or time-of-day methods [2]. Breakfast, brunch, lunch, dinner and supper EOs were classified as meals. Snack, morning/afternoon tea and beverage/break occasions were classified as snacks. Extended consumption EOs were classified as a meal or snack only if they occurred within 15 min of a preceding meal or snack, respectively [29]. The mean total frequency of all EOs, meals and snacks and the proportion of energy intake from meals and snacks were calculated.

### Participant characteristics

Information on respondents’ gender, age, highest education level, income, geographic region of residence, country of birth, employment status, number of hours worked in past week, social marital status and household composition was collected in the household survey [25]. Education level was categorised as: low (completed some high-school or less), medium (completed high-school or completed some high-school and/or certificate/diploma) or high (having a tertiary qualification). Participants’ weekly gross household income was provided by the ABS in deciles that took into account the number of persons living in the household [7]. These deciles were collapsed into quintiles and the reference ranges in Australian dollars per week were: quintile 1, <$398; quintile 2, $399–638; quintile 3, $639–958; quintile 4, $959–1438 and quintile 5, ≥ $1439. Geographic region of residence was categorised by the ABS as 1) Major cities of Australia 2) Inner regional cities of Australia 3) Other regions. Country of birth was categorised by the ABS as: Australia, other main English speaking countries and all other countries. Information on respondents’ labour force status (categorised as: employed, unemployed or not in the workforce) and the number of hours usually worked each week were used to classify participants as 1) Not in workforce/unemployed 2) <35 h 3) ≥35 h. Marital status was defined by the ABS as married (in a registered or de facto marriage) or not married. Household composition was categorised as 1) Person living alone 2) Couple only 3) Couple with children 4) One parent family with children 5) Unrelated persons aged ≥15 years 6) All other households. Due to small frequency counts in categories four to six, these were combined into one category, labelled “Other households”.

### Analytic sample

The analytic sample included adults ≥ 19 years who completed two 24 h recalls (*n* = 6053). In order to examine temporal eating patterns in the general population, participants were excluded if they were pregnant, breastfeeding, or had undertaken shift-work in the past four months (*n* = 687), due to their potential influence on how eating patterns are characterised. Participants were also excluded if they reported no energy intake during one of the 24 h recalls (*n* = 8), were missing information on the time an EO had commenced or had an EO that was either not identified as a meal/snack (e.g., EOs reported as other or don’t know [*n* = 116]). Therefore, the final analytic sample was 5242 adults (2402 men and 2840 women).

## Statistical analyses

### Latent classes of temporal eating patterns

Latent Class Analysis (LCA) was performed in M-*Plus* Version 7.31 (Muthen & Muthen, Los Angeles, CA, USA) to identify distinct temporal eating patterns for men and women, separately. LCA is a statistical technique that identifies categorical latent class variables on the basis of observed categorical variables [16]. For this study, energy intake within each hour of the day was examined and averaged across the two days of dietary recalls. Following this, binary variables indicating whether or not an EO providing a minimum energy content of ≥210 kJ had occurred within each hour were generated as the input variables for the LCA. A model with two latent classes was tested first and additional classes were added until the optimal number of latent classes was identified. Final class numbers were determined by 1) the evaluation of model fit indices, including the Akaike information criterion (AIC) and Bayesian information criterion (BIC), where smaller values indicate better model fit, 2) the Lo-Mendell-Rubin Likelihood Ratio test (LMR-LRT) and the Bootstrap Likelihood ratio test (BS-LRT) which compare *k* vs. *k* ^−1^ class models, where *k* is the number of latent classes and 3) pattern interpretability [31]. As there may be day-to-day variability in eating patterns (e.g., an EO may have been consumed at a certain time on the first recall day, but not on the second recall day), the reliability of the latent class solution was examined by repeating LCA procedures using data from one day of dietary recall.

### Associations between latent classes and sociodemographic and eating pattern indicators

All analyses for the associations of latent classes of EO timing with eating pattern variables and sociodemographic indicators were conducted in Stata 13 (Stata Inc., College Station, TX, USA). Person weights and replicate weights were applied to compute point estimates and standard errors to account for the probability of selection and the clustered survey design, respectively [25]. Descriptive statistics for sociodemographic and eating pattern variables are presented as weighted means (standard deviation) or weighted proportions. For continuous variables, the *F* test was used to determine differences between latent classes of EO timing with Bonferroni correction to account for multiple testing across >2 classes. For categorical variables, differences between latent classes were assessed using the adjusted Pearson Chi-2 test for survey data. For all analyses, *P* < 0.05 was considered statistically significant.

## Results

The mean (SD) ages of men and women were 47 (17) y and 49 (18) y, respectively (Table 2). The majority of men and women were born in Australia, had a medium education level, lived in major Australian cities and were married. Sixty percent of men reported usually working ≥35 h compared to 29% of women.

### Latent classes of temporal eating patterns

**Fig. 1:**
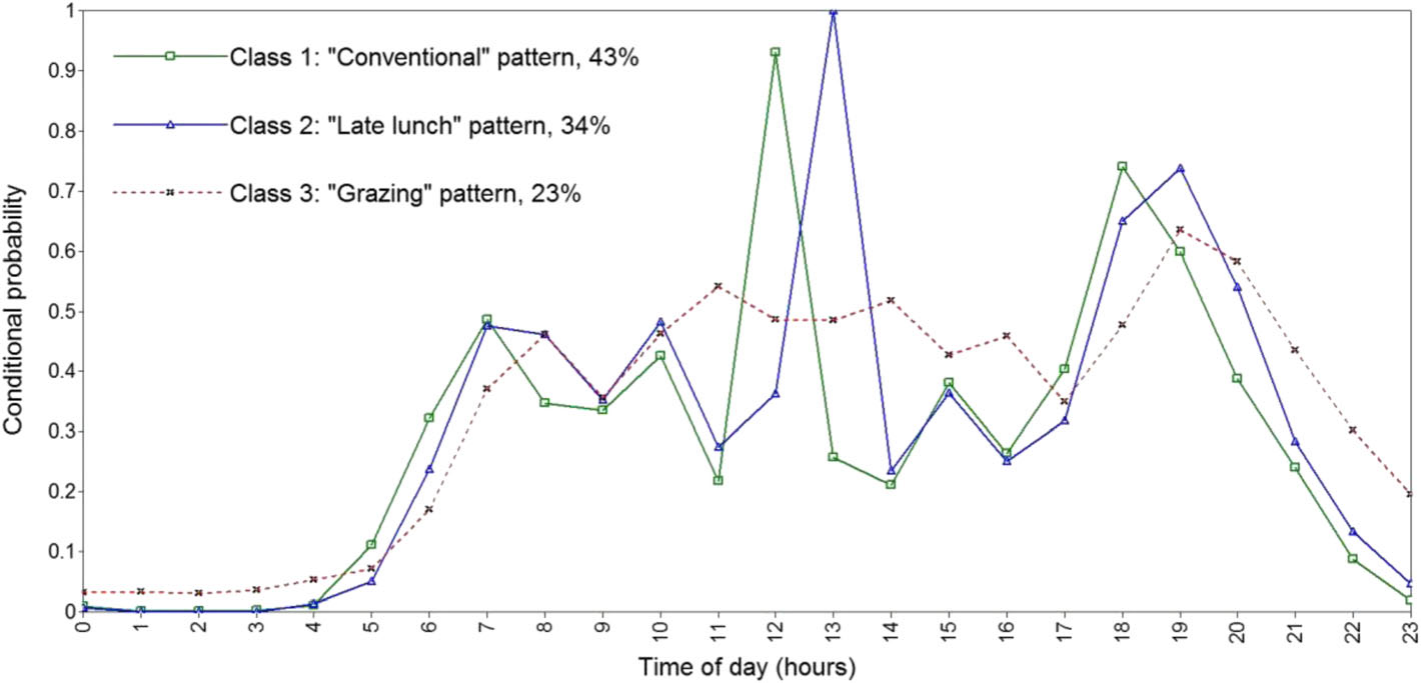
Conditional probabilities of eating occasion consumption across the day by latent class membership among Australian men. Lines with a *square* represent the “Conventional” temporal eating pattern. Lines with a *triangle* represent the “Later lunch” temporal eating pattern. *Dashed lines* with a cross represent the “Grazing” temporal eating pattern

**Fig. 2:**
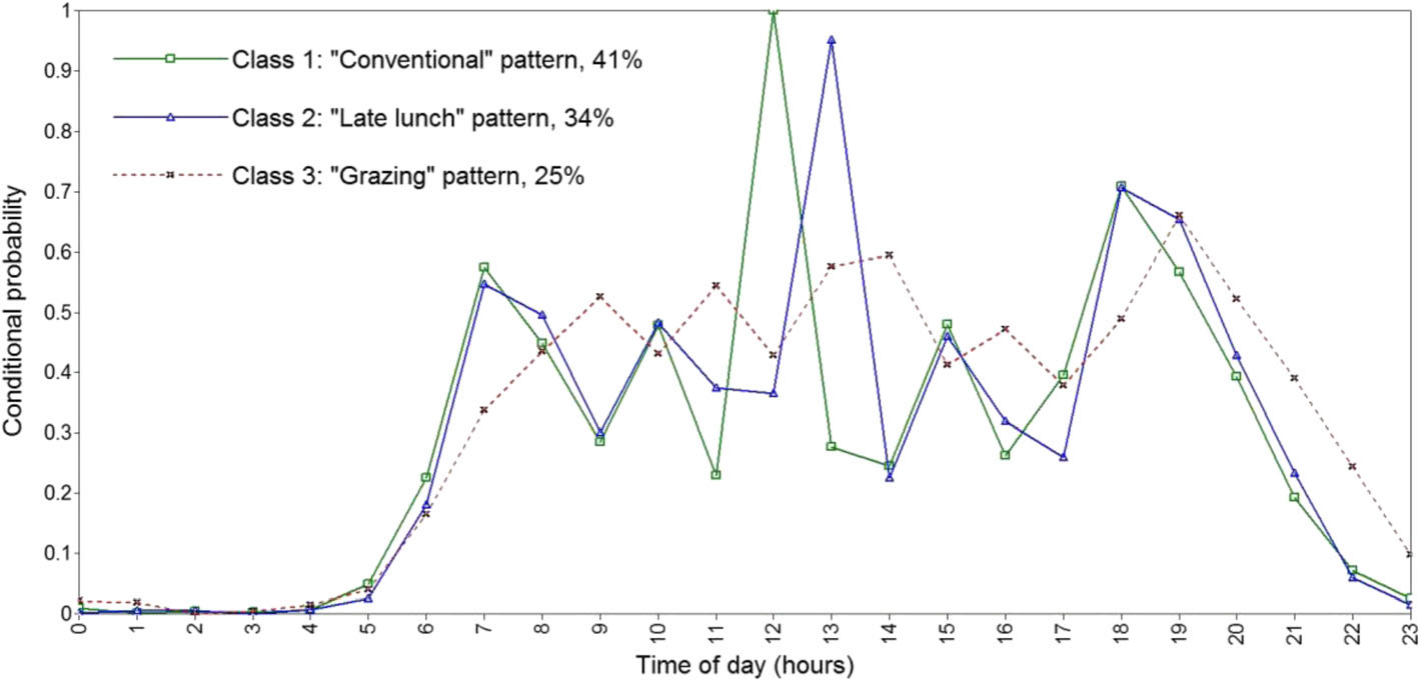
Conditional probabilities of eating occasion consumption across the day by latent class membership among Australian women. Lines with a *square* represent the “Conventional” temporal eating pattern. Lines with a *triangle* represent the “Later lunch” temporal eating pattern. *Dashed lines* with a cross represent the “Grazing” temporal eating pattern

Model fit indices favoured a three-class model for both men and women (Table 1). BIC and adjusted BIC values were smallest for the three-class solution and results from the LMR-LRT also favoured a three-class solution among men. The temporal eating patterns were descriptively similar in men and women (Figs. 1 and 2). Class labels were based on distinguishing features as shown by high or low conditional probability for consuming an EO at particular times of the day. The first pattern was labelled “Conventional” as participants in this class (43% men, 41% women) had a high conditional probability (>0.7) of consuming an EO at 12:00 and 18:00 h (e.g., conventional times in Australia for consuming the lunch and dinner meals). The second pattern, labelled “Later lunch” (34% men, 34% women) was characterized by having a high conditional probability (>0.9) of consuming an EO at 13:00 h (e.g., the lunch meal), one hour later than the “Conventional” pattern. Peaks in conditional probability of EO consumption between main meals was similar between the “Conventional” and “Later lunch” patterns, peaking at both 10:00 h and 15:00 h. The third pattern, labelled “Grazing” (23% men, 25% women), was characterized by frequent but no distinct peaks in probability of EO consumption (e.g., no peaks >0.7). The first peak in conditional probability of EO consumption occurred an hour later than the “Conventional” and “Later lunch” patterns. Additionally, conditional probabilities for consuming an EO after 20:00 h were also higher for the “Grazing” pattern compared to the other two patterns (Figs. 1 and 2). Similar temporal eating patterns solutions were found after repeating LCA procedures using one day of dietary recall (data not shown).

**Table 1:** Model fit indices for latent class models of temporal eating patterns^a^

	2 Classes	3 Classes	4 Classes	5 Classes	6 Classes
Men					
AIC	55869.734	55695.195	55568.741	55428.274	55556.894
BIC	56153.153	56123.215	56141.362	56145.497	56418.719
adjusted BIC	55997.469	55888.101	55826.817	55751.522	55945.313
LMR-LRT	−28139.701, *P* < 0.001	−27885.867, *P* = 0.03	−27773.598, *P* = 0.74	−27683.191, *P* = 0.07	−27592.446, *P* = 0.73
BS-LRT	−28139.701, *P* < 0.001	−27885.867, *P* < 0.001	−27773.598, *P* < 0.001	−27683.191, *P* < 0.001	−27592.446, *P* = 1.0
Women					
AIC	63452.109	63254.207	63163.371	62999.200	63103.155
BIC	63743.735	63694.623	63752.576	63737.193	63989.938
adjusted BIC	63588.045	63459.499	63438.018	63343.202	63516.513
LMR-LRT	−30624.734*, P* < 0.001	−31677.055, *P* = 0.017	−31553.104, *P* < 0.001	−31431.346, *P* = 0.07	−31347.200*, P* = 0.64
BS-LRT	−30624.734*, P* < 0.001	−31677.055, *P* < 0.001	−31553.104, *P* < 0.001	−31431.346, *P* < 0.001	−31347.200*, P* = 1.0

^a^ *AIC* Akaike Information Criterion, *BIC* Bayesian Information Criterion, *BS* Bootstrap, *LMR* Lo-Mendell-Rubin, *LRT* likelihood ratio test

### Sociodemographic profile of latent class

Table 2 presents the sociodemographic characteristics of Australian men and women, by latent class membership. Compared with the “Later lunch” and “Grazing” pattern, a higher proportion of men and women with a “Conventional” pattern had a lower education level (*P* < 0.001) and were either unemployed or not in the workforce (women only, *P* = 0.02). A higher proportion of women with the “Later lunch” pattern had a medium education level (*P* < 0.001) and were married (*P* = 0.01). Men and women with a “Grazing” pattern were significantly younger and a higher proportion were from major cities in Australia compared to those with a “Conventional” or “Later lunch” pattern (*P* < 0.01). A higher proportion of participants with a “Grazing” pattern also had higher education levels (women only, *P* < 0.001), a country of birth other than Australia/country where English is the main language spoken (women only, *P* < 0.001) and were not married (men only, *P* < 0.001). Conversely a significantly lower proportion of participants with a “Grazing” pattern lived in a couple household (men: *P* < 0.001, women: *P* = 0.01) or reported usually working ≥35 h per week (men only, *P* = 0.03), when compared to the other two classes.

**Table 2:** Sociodemographic characteristics of temporal eating patterns, overall and by latent class membership

	Men (*n* = 2402)					Women (*n* = 2840)				
	Overall	Conventional	Later Lunch	Grazing	*P* value^1^	Overall	Conventional	Later Lunch	Grazing	*P* value^1^
Age (y, mean [SD])	47 (17)	51 (17)^a^	48 (16)^b^	41 (16)^c^	<0.01	49 (18)	51 (20)^a^	50 (16)^a^	46 (17)^b^	<0.001
Education level (%)					<0.001					<0.001
Low	21	26	17	17		29	35	26	25	
Medium	54	54	54	53		42	41	47	39	
High	26	21	30	30		28	24	27	36	
Weekly income (quintiles, [%])^2^					0.14					0.24
First: <$398	16	18	13	18		22	25	19	21	
Second: $399–638	17	17	17	16		19	19	19	18	
Third: $639–958	19	21	19	17		19	20	19	19	
Fourth: $959–1,438	23	23	21	23		19	18	18	22	
Fifth: ≥ $1439	25	21	30	26		21	18	25	20	
Geographic region (%)					<0.01					<0.01
Major cities of Australia	73	69	72	81		74	70	74	80	
Inner regional cities	18	20	21	12		18	20	19	13	
Other areas (rural or remote)	9	11	8	7		9	10	7	7	
Country of birth (%)					0.23					<0.001
Australia	69	70	71	63		69	71	74	59	
Main English speaking countries	12	12	11	14		12	12	12	12	
All other countries	19	18	18	23		19	17	15	29	
Hours worked in past week (%)					0.03					0.02
Not in workforce	30	31	26	33		44	49	42	39	
< 35 h	10	8	11	14		27	24	30	28	
≥ 35 h	60	61	63	53		29	28	28	33	
Social marital status (%)					<0.001					0.01
Married^*3*^	63	67	67	48		59	58	64	56	
Not married	37	33	33	52		41	42	36	44	
Household composition (%)					<0.001					0.01
Person living alone	13	14	11	15		16	19	14	15	
Couple only	32	36	32	23		31	31	34	26	
Couple with children	38	35	42	38		34	30	26	27	
Other household	17	15	15	24		19	20	17	22	

^1^Different superscript letters indicate significant differences between classes, assessed using a F-test with Bonferroni correction for continuous variables. Differences between classes for categorical variables were assessed using an adjusted Pearson Chi2 test

^2^ *n* = 2258 men and *n* = 2586 women due to missing cases for income

^3^In a registered or de facto marriage

### Eating pattern profile of latent classes

Differences in eating pattern characteristics according to latent class membership are presented in Table 3. Among men only, the “Conventional” pattern had a lower EO and snack frequency and lower total energy intakes but a longer time between EOs, when compared to the “Later lunch” and “Grazing” patterns. However, the proportion of energy intake from snacks did not differ significantly from the “Later lunch” pattern among men. Compared to the “Conventional” and “Later lunch” patterns, those with a “Grazing” pattern had a higher EO frequency (men: *P* < 0.05, women: *P* < 0.001), snack frequency (men: *P* < 0.05, women: *P* < 0.001), a higher total energy intake (men: *P* < 0.01, women: *P* < 0.05) and a higher proportion of total energy intake from snacks (*P* < 0.001). The “Grazing” pattern also had a significantly lower meal frequency (men only, *P* < 0.01), a lower proportion of total energy intake from meals (*P* < 0.001) and shorter time between EOs (women only, *P* < 0.01)

**Table 3:** Eating pattern characteristics of temporal eating patterns, by latent class membership. Results are means (SD)^1^

	Men				Women			
	Conventional	Later Lunch	Grazing	P for trend	Conventional	Later Lunch	Grazing	P for trend^2^
EO frequency	4.6 (1.2)^a^	4.8 (1.1)^b^	5.2 (1.3)^c^	<0.05	4.6 (1.2)^a^	4.7 (1.1)^a^	5.1 (1.3)^b^	<0.001
Meal frequency	2.9 (0.5)^a^	3.0 (0.5)^a^	2.8 (0.6)^b^	<0.01	3.0 (0.5)	3.1 (0.5)^a^	3.0 (0.6)^b^	<0.05
Snack frequency	2.3 (1.3)^a^	2.5 (1.3)^b^	2.9 (1.4)^c^	<0.05	2.5 (1.5)^a^	2.6 (1.3)^a^	3.1 (1.5)^b^	<0.001
Time between EOs (min)	220 (69)^a^	211 (62)^b^	205 (68)^b^	<0.05	212 (67)^a^	205 (67)^a^	192 (62)^b^	<0.01
Total EI (kJ)	9085 (2777)^a^	9556 (2710)^b^	10456 (3122)^c^	<0.01	7012 (2398)^a^	7320 (2415)^a^	7661 (2487)^b^	<0.05
Total EI from meals (%)	79 (15)^a^	79 (13)^a^	71 (15)^b^	<0.001	80 (13)^a^	79 (13)^a^	73 (15)^b^	<0.001
Total EI from snacks (%)	21 (14)^a^	21 (13)^a^	29 (15)^b^	<0.001	20 (13)^a^	21 (13)^a^	27 (15)^b^	<0.001

^1^ *EI* energy intake, *EO* eating occasion

^2^Different superscript letters indicate significant differences between classes, assessed using a F-test with Bonferroni correction

## Discussion

Using LCA as a novel approach, this study examined temporal eating patterns across the day and cross-sectional associations with sociodemographic and eating pattern indicators among a nationally representative sample of Australian men and women. We believe this is one of the few studies, among adults, to examine temporal eating patterns using information on EOs consumed across the day [13]. Three distinct temporal eating patterns were found among both men and women, each with a different sociodemographic and eating pattern profile.

The finding of different temporal eating patterns that varied by sociodemographic and eating pattern characteristics highlights the complexity of eating pattern behaviors and the utility of exploratory, data-driven methods to objectively identify temporal eating patterns that may not have been detected if based on *a priori* and arbitrary time-of-day approaches. Only one recently published study has examined temporal eating patterns [14]. Using kernel k-means cluster analysis, Eicher-Miller et al. [13] found four patterns among US adults based on participant proportional energy intake and time and frequency of EO consumption. One of these patterns was characterized by frequently peaked consumption of hourly proportional energy intake. This pattern is similar to the “Grazing” pattern found in the present study. Other studies conducted in Finnish [32], French [23] and US adults [33] also support the finding of a less conventional eating pattern that doesn’t consist of three meals per day. In another study, eating on the run was also reported by 50–60% of young US adults (*n* = 1687) suggesting disruptions to meal regularity are not uncommon.

In this study, both men and women with a “Grazing” pattern were younger and had a higher EO and snack frequency than the “Conventional” or “Later lunch” patterns. The consumption of the first and last EOs occurred later in the day, suggestive of a later, but more frequent EO pattern. Total energy intake and the proportion of total energy intake from snacks was also higher. Previous research has shown that later timing of the last EO [34], consuming a higher proportion of total energy intake after 17:00 h [11, 12] or at the dinner meal [35] and having a higher evening to morning energy intake ratio [36] is positively associated with higher overall energy intakes. However, the metabolic implications of this later eating pattern are unclear, and future research should examine the relationship between temporal eating patterns and health.

In the present study, two temporal eating patterns were characterized by three distinct times of peak EO consumption (typically when main meals are consumed in Australia), and were differentiated only by the timing of the second EO peak (e.g., the lunch meal). Differences in temporal eating patterns may be influenced by contextual (e.g., timing of the work lunch break) and sociodemographic factors (e.g., household composition/employment) that can affect the routine and structure of the day [21, 23]. For example, compared to the other patterns, a lower proportion of men with a “Grazing” pattern worked full-time and were married. In contrast, a higher proportion of women with a “Grazing” pattern worked full-time and had a higher education level. Furthermore, a higher proportion of men and women with the “Later Lunch and Conventional patterns were from couple only households.

The sociodemographic characteristics of temporal eating patterns have rarely been investigated [13]. A relationship between a later, more frequent EO pattern and being non-Hispanic Black or being from a low-income household was reported by Eicher-Miller et al. [13]. In the present study, no association between temporal eating patterns and income was found and, among women only, the “Grazing” pattern was associated with a higher education level and being born in a non-English speaking country. These findings indicate that the sociodemographic profiles of temporal eating patterns are complex and differ for men and women. Future research utilizing real-time data on lifestyle habits (e.g., sleep, work, physical activity patterns) and context (e.g., location, EO duration, people present) is needed to better understand the factors that influence temporal eating patterns.

To date, few studies have investigated temporal eating patterns and the approaches used to assess them also widely vary [4]. This is probably, in part, due to limitations in the dietary assessment methods (e.g., questionnaires) often used in observational studies and the lack of understanding of statistical techniques that can capture and analyze the complexity of eating patterns across the day. The statistical approach taken may also depend on which dimension of temporal eating patterns is being examined (e.g., frequency, timing or regularity) and whether the focus is on EOs or energy intakes. For example, the present study used LCA for categorical data to assess timing and frequency of EOs across the day, whereas Eicher-Miller et al. [13] used cluster analysis for continuous data to assess timing and frequency of hourly proportional energy intake across the day. However, in both studies, temporal eating patterns were only based on one to two days of dietary recall, and therefore may not capture the day-to-day variation in temporal eating patterns. It is unknown if these patterns are stable over the long term or whether patterns differ on weekend versus weekdays and this is an area that warrants further research.

A limitation of the present study is that temporal eating patterns were derived using a person-centred, data-driven method and findings may therefore not be generalizable to populations from other countries. Little is known about the accuracy of self-reported eating patterns. The eating patterns in this study were assessed using 24-h dietary recall data which is prone to recall bias and underreporting. In a recent study of 83 adults, it was estimated that a mean total energy intake of ~30% was underreported by 20% of participants in the 24-h recall when compared to energy expenditure estimated using the Doubly Labelled Water method [37]. There is also some evidence that energy underreporting is associated with irregular meal habits [37] and the underreporting of EO frequency [38]. Research is needed to better understand the accuracy of self-reported eating patterns. Meals and snacks were also classified based on participant-identification of EOs which involves subjectivity in participants’ allocation of an EO as a meal or snack. The researcher must also decide how to treat EOs that are not clearly defined as a meal or snack (e.g., supper).

Strengths of this study include the large, nationally representative sample of Australian men and women and the use of a more objective EO definition based on previous research [29, 30]. A novel feature of the study was the objective approach using LCA to examine temporal eating patterns while taking into consideration EOs consumed across the day. Standardised criteria were used to determine the number of clusters which minimises reliance on researchers’ preconceived notions of meal times.

## Conclusion

In conclusion, using LCA as a novel approach, three distinct temporal eating patterns that differed by sociodemographic and eating pattern profiles were identified among a representative sample of Australian men and women. LCA may therefore be a useful approach to capture the timing of EOs, including meals and snacks, across the day. However, future research is needed to examine whether these temporal eating patterns are associated with health outcomes.

## Abbreviations

ABS: Australian Bureau of Statistics
AIC: Akaike information criterion
BIC: Bayesian information criterion
BS-LRT: Bootstrap likelihood ratio test
EO: Eating occasion
LCA: Latent class analysis
LMR-LRT: Lo-Mendell-Rubin likelihood ratio test
NNPAS: National nutrition and physical activity survey
